# Research on the pathways to enhance industry-academia-research institute collaboration and innovation: from the perspective of subway opening

**DOI:** 10.3389/frma.2026.1752085

**Published:** 2026-05-18

**Authors:** Qianchun Dai, Li Yuanheng, Chengsheng Tong

**Affiliations:** 1 Accounting Department, Shanghai National Accounting Institute, Shanghai, China; 2 School of Information, Central University of Finance and Economics, Beijing, China

**Keywords:** digital transformation, enterprise cooperation culture, high-quality talents, industry-academia-research cooperation, infrastructure construction

## Abstract

As a crucial component of urban transportation infrastructure, the subway not only optimizes the urban transportation network but also accelerates the flow and aggregation of innovative resources, holding significant importance for enhancing the level of Industry-academia-research cooperation among enterprises. This study utilizes panel data from A-share listed companies on the Shanghai and Shenzhen stock exchanges from 2010 to 2022, employing a multi-time point difference-in-differences method to delve into the promotional effect of subway opening on enterprise Industry-academia-research cooperation. The findings reveal that the opening of a subway significantly elevates the level of Industry-academia-research cooperation among enterprises, and this conclusion remains valid after a series of robustness tests. Mechanism analysis indicates that subway opening primarily promotes enterprise Industry-academia-research cooperation through three pathways: enhancing corporate cooperation culture, increasing the proportion of high-quality talent in enterprises, and elevating the level of digital transformation. Heterogeneity analysis shows that the promotional effect of subway opening on the level of enterprise Industry-academia-research cooperation is more pronounced in non-state-owned enterprises, high-tech enterprises, and small-scale enterprises. The research conclusions provide reliable empirical support for the promotion of enterprise Industry-academia-research cooperation by subway opening and offer insights and references for the government to further advance decision-making related to transportation infrastructure construction and high-quality enterprise development.

## 1 Introduction

Since the twenty-first century, countries worldwide have invested heavily in the construction of transportation infrastructure. As the cornerstone of urban development, transportation infrastructure not only bears the crucial task of transporting people and goods but also serves as a key driver for economic growth and comprehensive social progress. As of 2023, the total length of urban rail transit globally has reached 43,400.39 km, with China ranking first in the world with a mileage of 11,900.29 km. Currently, China has 306 urban rail transit lines operating in 55 cities, with an annual passenger volume of 29.44 billion, representing an increase of approximately 50% compared to the previous year (He et al., 2024). China's urban rail transit system ranks first in the world in terms of operational mileage, number of stations opened, and number of lines. Among them, the subway, with its unique advantages of high capacity, efficiency, and low pollution, has become an indispensable core component of China's urban rail transit system, profoundly transforming residents' travel patterns. The subway has not only significantly alleviated urban traffic congestion, improved travel efficiency for residents, and promoted economic development and social prosperity in areas along the lines but has also subtly increased the frequency of exchanges between enterprises and universities and research institutions, deepening Industry-academia-research collaboration.

The significant economic and social value demonstrated by the modern transportation facility of the subway has become a focal point of in-depth academic research today. Scholars argue that the opening of subways enriches residents‘ travel options and reduces their travel costs at the economic level, exerting a substantial impact on urban economic development (Zhang, 2020). Specifically, the opening of subways enables central city businesses to hire labor from the urban periphery on a large scale and enhances their mobility and productivity (Haddad et al., 2015), effectively driving employment growth (Mayer and Trevien, 2017). Simultaneously, the opening of subways promotes increases in land and housing prices, injecting new vitality into the sustained development of the urban economy (An et al., 2024). At the social level, the construction and operation of subways necessitate a focus on optimizing construction plans to minimize negative impacts on residents' daily lives. Subways play a crucial role in alleviating urban traffic congestion (Gu et al., 2021) and reducing air pollution levels (da Silva et al., 2012). Furthermore, as a rapid, convenient, highly accessible, and cost-effective mode of travel, subways bring significant benefits to urban tourism (Cui et al., 2024). The opening of subways is a hallmark of metropolises, significantly enhancing urban attractiveness and competitiveness (Bono et al., 2022), and synergizing with economic benefits to promote urban development. However, existing research predominantly focuses on the economic and social utilities of subway openings, with relatively scarce studies on how subways influence corporate innovation activities from a micro perspective, particularly the impact on Industry-academia-research collaboration at the enterprise level.

The scale of Industry-academia-research cooperation in China's economic and social development continues to expand, this is a cooperative innovation activity among enterprises, universities and scientific research institutions, which can significantly increase the technological innovation achievements of the partners. By the end of 2023, the total contract value for the transformation of scientific and technological achievements of universities and research institutes nationwide reached 28.6 billion USD, an increase of 13.7% year on year. Enterprise-led integration of Industry-academia-research collaboration is an effective way to improve the efficiency of the innovation system (Chen and Ye, 2025), and continuously strengthen the competitiveness of industrial development (Zeng et al., 2023). However, there are still many problems in the process of Industry-academia-research collaboration. In particular, the collaboration mechanism has not fully embodied the driving role of technological progress in economic development, which requires further improvement in the depth, breadth and closeness of collaboration (Xu et al., 2023). Moreover, regional disparities in resource allocation directly affect the efficiency of cooperation (Liu et al., 2020). A particularly sensitive issue arises from geographical distance, as many universities and research institutions are often located in suburban or newly developed areas, far from enterprise-dense regions. This physical distance increases the cost of collaboration and restricts the flow of knowledge and the sharing of resources. Taking Shanghai as an example, Yangpu University Town is about 15 km away from the enterprise cluster in the central business district, Songjiang University Town is about 30 km away, and Lingang University Town is up to 75 km away. Similar phenomena of large distances between enterprises and university or technology park clusters are common in other cities, which to some extent constrains the efficient implementation of Industry-academia-research collaboration and affects the rapid transformation of innovation outcomes (Tang et al., 2020). The different participants in Industry-academia-research collaboration are often distributed across various regions of the city, making the efficiency of their offline exchanges contingent upon the operational efficiency of urban transportation infrastructure. According to theoretical analysis based on the knowledge spillover model, reducing spatial distance facilitates increased opportunities for face-to-face exchanges among different entities, particularly enhancing the transmission efficiency of tacit knowledge (Brucks and Levav, 2022). The opening of subways provides a superior platform for enterprise Industry-academia-research collaboration.

In light of this, this paper aims to explore the specific impact of subway opening on companies‘ Industry-academia-research collaborations by focusing on collaborative patents. Taking Chinese A-share and Shenzhen-listed companies from 2011 to 2022 as the research sample, this paper employs a multi-period difference-in-differences model to empirically analyze the relationship between subway opening and Industry-academia-research collaboration innovation. The research results show that the opening of subways significantly increases the number of Industry-academia-research collaboration patents for enterprises, mainly by improving the corporate culture of collaboration, increasing the proportion of high-quality talents, and raising the digitalization level of enterprises, thereby significantly improving the level of Industry-academia-research collaboration innovation among enterprises along the subway lines. Further heterogeneity analysis reveals that the opening of subway lines has a more pronounced effect on enhancing Industry-academia-research collaboration innovation for non-state-owned enterprises, high-tech enterprises, and small enterprises. This finding not only sheds light on the different effects of subway opening on enterprises' innovation activities in different scenarios, but also provides policy implications for optimizing the layout of urban infrastructure and promoting deep integration of Industry-academia-research collaboration.

The marginal contributions of this paper are mainly manifested in the following three aspects: First, this paper is the first to integrate the subway opening and enterprise innovation in Industry-academia-research collaboration into a unified analytical framework, revealing the facilitating effect of subway opening on innovation activities from the micro-enterprise level. This perspective goes beyond the traditional analytical framework of economic and social effects of subways in research, provides new insights into the cross-integration of enterprise innovation theory and transport economics theory, and provides systematic evidence for accurately understanding the close relationship between subway opening and enterprise Industry-academia-research collaboration. Second, this paper enriches the research literature on the innovative role of subway opening, especially in the area of how infrastructure construction affects enterprise-Industry-academia-research collaboration. Through empirical analysis, this paper not only confirms the positive impact of subway opening on enterprise-Industry-academia-research collaboration, but also explores the underlying mechanisms of this impact, namely, the promotion of enterprise-Industry-academia-research collaboration through the improvement of collaboration culture, the agglomeration of high-quality talents, and the enhancement of enterprise digitalization level, thus expanding the research frontiers in related fields and providing solid empirical evidence for the promotion of enterprise-Industry-academia-research collaboration. Third, this paper explores the influence of enterprise heterogeneity on the impact of metro opening, incorporating factors such as whether an enterprise is state-owned, whether it is a high-tech enterprise, and the size of the enterprise into the analytical framework, thereby revealing the heterogeneous characteristics of the impact of metro opening on enterprise innovation.

The remainder of this paper is organized as follows: The second section presents the literature review, the third section outlines the theoretical framework and research hypotheses, the fourth section details the research design, the fifth section conducts empirical analysis, and the sixth section presents the research conclusions and policy recommendations.

## 2 Literature review

The present chapter offers a summary and review of the extant literature pertinent to the research question, systematically organizing it according to the economic, social and innovation benefits generated by the opening of subways. It then clearly identifies the existing achievements and research gaps concerning the innovation benefits brought about by subway openings, thereby pointing out the direction for further research.

The opening of subterranean rail networks exerts a multifaceted influence on urban economies, not only reducing citizens' travel expenditures and enhancing transportation efficiency but also optimizing the allocation of urban resources and fostering the vigorous development of industries along the lines. Excessive traffic congestion has emerged as a major challenge in global urban development today (Haywood et al., 2018). A substantial body of research has demonstrated that public transportation facilities can effectively address this issue. When the public transit system is assumed to cease operation, traffic delays increase by 47% (Anderson, 2014). Among these, subways, with their unique advantages of speed, punctuality, and high capacity, significantly alter residents' choices in travel modes (Jones, 2017; Li Z. et al., 2026; Li X. et al., 2026). Specifically, the introduction of a new subway line has been shown to divert approximately 4.1% of bus passengers annually (equivalent to saving at least 10 bus routes). It is noteworthy that subways complement the existing public transportation system without exerting a crowding-out effect on the number of buses or the length of bus routes (Liu and Li, 2020), In some cities, 1 year after the opening of the subway, the rush hour speed of the nearby roads greatly increased (Tang et al., 2025), and the time value of commuting was saved by 0.1 US dollars for each car or bus (Gu et al., 2021) thus demonstrating the synergistic utility between subway openings and the city's existing public transportation system. Furthermore, the extensive coverage of the subway network within the city enhances the accessibility of the public transportation system (Yu and Cui, 2023). Conversely, the efficacy of subways in enhancing residents' travel quality is attributable to their punctuality, safety, and substantial transport capacity. The daily commute by subway is estimated to reduce commuting costs by approximately 60%, while also increasing travel efficiency by an estimated 30 min (Reddy et al., 2010).

The opening of subway lines has been shown to have significant positive effects on the surrounding areas, often resulting in increased land value and improved real estate market conditions, which in turn stimulates the growth of related industries, such as commerce and services. At the same time, it enhances the mobility of labor force, improves urban productivity and promotes the utilization of urban underground space (UUS) to adapt to urban functions (Lin et al., 2022). Research indicates a significant positive correlation between the density of subway stations within a city and economic growth, with every 10% increase in subway station density contributing 0.054% to annual GDP growth, particularly in large cities with populations exceeding 6.15 million and high transportation demand (Zhang, 2020). From a land value perspective, the introduction of subways has been shown to increase the average land value along the lines by approximately 20%−30%, thereby indirectly promoting the prosperity of the real estate market (Bae et al., 2003). The premium effect of urban rail transit on real estate has been demonstrated to attract high-income individuals to reside along subway lines, thereby attracting high-end retail and dining establishments to cluster nearby (An et al., 2024). The development of these industries has been shown to provide more market opportunities for enterprises along the lines and even in suburban economies (Tao et al., 2023). Furthermore, the opening of subterranean railways will effect a new urban direction, with land use intensity around stations exhibiting a marked increase in comparison to other areas (Han and Wu, 2023), and a 30% increase in the number of new enterprises around stations following the opening of the subway (King, 2011). As the subway network undergoes continuous improvement, the city undergoes gradual evolution from a single-core to a multi-core development pattern, with areas surrounding subway stations becoming new urban growth poles (Xia and Han, 2020). This enhancement in land use efficiency and optimization of urban spatial structure attracts enterprises and talent to cluster in areas along the lines, forming new industrial agglomeration zones (Yan et al., 2020). The opening of subways is also of great significance in optimizing the allocation of urban resources. Metropolises with subways have been shown to enhance their overall image and competitiveness (Bono et al., 2022), and to demonstrate stronger advantages in attracting investment and talent (Wei et al., 2024). The areas adjacent to subway lines often evolve into talent highlands and innovation centers within the city, thereby providing continuous momentum for the city's long-term development (Wei et al., 2024). Moreover, as a highly accessible and economical mode of travel, subways significantly influence tourists' experiences of urban attractions (Cui et al., 2024). The enhancement of the subway network facilitates seamless transit, thereby promoting the vigorous development of the tourism industry (Cui et al., 2024). Moreover, this progression indirectly drives the optimization and upgrading of the industrial structure, providing substantial support for the diversified development of the urban economy (Wei et al., 2024). In conclusion, the construction of subways has become a pivotal element of contemporary urban planning and development, playing a crucial role in enhancing urban competitiveness and promoting sustainable and robust economic growth.

The social benefits engendered by the introduction of subterranean rail networks are manifold and wide-reaching, effecting positive contributions to the enhancement of residents' wellbeing, employment generation and the promotion of sustainable environmental development. The introduction of subterranean rail networks has been demonstrated to be an effective measure in alleviating urban traffic congestion and enhancing the efficiency of urban transportation systems. This transformation not only directly reduces the enormous economic costs associated with traffic congestion but, more importantly, fundamentally enhances residents' quality of life and happiness, particularly in developing countries that are in the midst of rapid development. The construction and operation of subways has become a significant indicator of improved urban wellbeing (Yin et al., 2021). The proximity of residences to subway stations has been found to be positively correlated with residents' happiness, indicating that those living closer to subway stations experience stronger feelings of happiness (Li et al., 2018). The opening of subways has been shown to foster a sense of interconnectedness, not only by reducing physical distances but also by fostering emotional and psychological connections among individuals (Chen et al., 2024). This, in turn, has the potential to create a more positive and efficient communication atmosphere on an emotional level. Concurrently, subways furnish residents with access to enhanced healthcare services, thereby significantly improving their health status and reducing the prevalence of diseases. This phenomenon is particularly pronounced among high-income groups, middle-to-lower age groups, and those with a moderate level of education (Chen et al., 2024). The opening of subways directly drives employment growth. The enhanced connectivity of rail transit facilitates labor mobility between central urban areas and peripheral regions, enabling enterprises to recruit at scale while effectively leveraging suburban workforce resources. This dynamic elevates overall productivity and operational flexibility, resulting in an approximate 8.8% increase in aggregate urban employment (Haddad et al., 2015). The thriving commercial districts around subway stations offer residents diverse business opportunities and also attract a large number of professionals to specialized fields such as shopping malls and financial centers, promoting further optimization of the city's human resource allocation. In terms of environmental protection, subways generate significantly lower carbon emissions during operation compared to traditional transportation modes like cars, playing a crucial role in mitigating urban pollution issues (Mayer and Trevien, 2017). In numerous developing economies across China and globally, the expansion of subway systems has become a key strategy for addressing air pollution and traffic congestion problems (da Silva et al., 2012). Statistical evidence demonstrates that the introduction of subways can lead to substantial enhancements in air quality. For cities with initially high pollution levels, the launch of subways can reduce particulate matter by 4% in the surrounding areas of city centers (Gendron-Carrier et al., 2022) and enhance health benefits from reduced morbidity (Li et al., 2019). Vigorously building the subway system has a good inhibitory effect on air pollutants and is an important starting point for regional sustainable development (Lin et al., 2025). However, it is important to note that an indiscriminate increase in subway mileage, lines, and stations may have a marginal adverse effect on air quality levels. Policymakers are therefore advised to focus on actively advocating for green transportation and reasonably planning the subway system network (Xiao et al., 2020).

Despite the proliferation of research in recent years on the impact of subways on economic and social development, literature that delves into the innovative benefits brought about by the opening of subways remains relatively scarce. When examining the effects of subway openings from the perspective of innovative collaboration, the importance of communication, as a fundamental aspect of cooperation, becomes evident. In the contemporary digital era, while online communication is undoubtedly convenient, it is limited by its inability to fully guarantee the authenticity and effectiveness of information transmission, and may even inhibit the generation of innovative ideas due to the lack of immediate feedback and deep interaction. Consequently, offline communication emerges as a crucial avenue for compensating for the shortcomings of online communication and enhancing cooperation efficiency (Brucks and Levav, 2022). The knowledge spillover model posits that reducing spatial distance fosters increased opportunities for face-to-face interactions among different entities, particularly enhancing the efficiency of tacit knowledge transfer. Statistical analysis indicates that the introduction of a subway line results in a substantial increase in the number of collaborative patents, with a range of 14.85% to 37.69% (Koh et al., 2022). Moreover, the introduction of subways has been shown to reduce commuting costs for enterprises, thereby encouraging them to allocate more funds to research and development expenses. This is regarded as a forward-looking strategy for future market competitive advantages and provides robust momentum for achieving high-quality development of enterprise innovation capabilities. The opening of subways optimizes the communication environment, accelerates knowledge spillovers, and incentivizes R&D investments, constructing a highly efficient system for collaborative innovation and cooperation of profound value (Wei et al., 2024).

The impact of subway openings on urban economic, social, and innovative dimensions is multifaceted and profound. However, extant research demonstrates deficiencies in examining the influence of subway openings on enterprise, Industry-academia-research collaboration at the micro level. Firstly, there is a paucity of studies that specifically investigate the impact of subway openings on such collaboration within a cohesive analytical framework. Secondly, as inferred from the aforementioned analysis, the enhancement of innovation efficacy through subway openings may exert a more pronounced effect in developing countries. As the nation with the largest output of Industry-academia-research collaboration among developing countries, China is highly representative. Previous research has predominantly focused on the influence of inter-regional transportation infrastructure development on innovation cooperation between two locations, with a scarcity of literature examining the impact of improving intra-regional transportation infrastructure on enterprise innovation collaboration. The limited studies that have explored the effects of enhancing intra-regional transportation facilities have mostly concentrated on a single city or enterprise, with results potentially biased due to unobservable individual-specific fixed effects, and none have delineated innovation capability down to the Industry-academia-research collaboration indicator. Different from the traditional linear regression model, the double difference model alleviates endogenous bias and provides reliable causal inference by dealing with counterfactual analysis of the control group and the control group. The study period was chosen from the pilot to the large-scale promotion of subway, when the number of cities opened increased and the sample expanded, which improved the estimation accuracy and universality.

Thus, In order to address the deficiencies at the empirical level, this paper employs a sample of Chinese A-share and Shenzhen-listed companies from 2011 to 2022 in order to construct micro-panel data. Employing a multi-time-point difference-in-differences approach, it explores the impact and mechanisms of subway openings on enterprise Industry-academia-research collaboration, and delves deeper into this influence by considering the nature of enterprises. The research findings of this paper offer a novel perspective centered on enterprise Industry-academia-research collaboration for an in-depth exploration of the benefits of subway openings. The findings of the study provide a theoretical reference point for governments to optimize the layout of infrastructure development, offer practical guidance for enterprises to develop their own Industry-academia-research collaboration, and serve as a basis for the entire society to strengthen innovation cooperation, promote economic development, and enhance social welfare.

## 3 Theoretical analysis and research hypothesis

Transportation infrastructure, the lifeblood of urban economic development, directly influences the efficiency and effectiveness of corporate innovation activities based on its level of development (Heblich et al., 2020). As a core component of modern urban rail transit, according to the triple helix theory (Leydesdorff and Etzkowitz, 1998), the collaborative interaction among government, universities and enterprises is the core driving force of innovation ecosystem. Subway construction is essentially the government-led supply of infrastructure public goods, and its planning and layout not only reflects policy orientation, and subways significantly reduce transportation costs between enterprises and innovation entities (Gu et al., 2021). This cost reduction not only facilitates the flow and integration of innovation elements but also provides enterprises with broader markets and innovation resources. According to the theory of new economic geography (Coughlin and Segev, 2000), decreased transportation costs help optimize the layout of economic activities within cities, enabling enterprises and universities to interact more conveniently. Firstly, when seeking collaborators, researchers often face a trade-off between communication costs and the suitability of potential partners. Geographical distance is a primary obstacle, with greater distance reducing the likelihood of collaboration (van der Wouden and Youn, 2023). Consequently, enterprises tend to collaborate with local universities to minimize search costs (Catalini et al., 2020). The opening of subways, by shortening physical distances and reducing communication costs, offers an efficient communication medium for deep collaboration between enterprises and innovation entities such as universities (Alzamora-Ruiz et al., 2021), promoting information sharing, knowledge exchange, and technical cooperation, thereby enhancing the level of enterprise Industry-academia-research collaboration. Secondly, the theory of collaborative innovation emphasizes the optimal allocation and efficient utilization of innovation resources (Vivona et al., 2023). Subway networks enable enterprises to more easily access the latest scientific research achievements and technical information, complementing the resource advantages of universities and promoting the expansion of Industry-academia-research collaboration into more fields. However, information asymmetry is a key factor constraining the in-depth development of Industry-academia-research collaboration (Liang et al., 2023). Due to the different divisions of labor between universities and enterprises, resource differences lead to asymmetric information distribution. The opening of subways provides a more confidential and reliable efficient channel for offline collaboration, effectively alleviating this issue and improving the quality of innovation cooperation outcomes (Brucks and Levav, 2022). Furthermore, from the perspective of knowledge spillover effects, subways facilitate the rapid dissemination of knowledge and information within cities. Technical knowledge and information exhibit strong positive externalities (Cui and Li, 2022), and universities' research and innovation generate knowledge spillovers, increasing the likelihood of exchanging innovative ideas with enterprises and stimulating their enthusiasm for innovation collaboration (Fan et al., 2023). Subway networks enable enterprises to more easily access university knowledge resources, deepening and expanding Industry-academia-research collaboration. Lastly, the opening of subways also brings about industrial agglomeration effects, optimizing the layout of urban internal industrial distribution and resource allocation. Improving transportation facilities significantly enhances location attractiveness, attracting enterprises to cluster around transportation hubs or along subway lines (Tsivanidis, 2019), further promoting Industry-academia-research collaboration among enterprises along subway lines and eliminating geographical distance concerns when considering collaboration between enterprises and universities. In summary, by shortening geographical distances, promoting information exchange, and fostering innovation diffusion, the opening of subways enhances the level of enterprise Industry-academia-research collaboration, optimizes the urban internal innovation environment, and injects new vitality into enterprise innovation development and industrial upgrading. Based on the aforementioned theoretical analysis, we propose Hypothesis 1.

Research hypothesis one: The opening of a subway system can enhance the level of Industry-academia-research collaboration among enterprises.

The opening of the subway system has optimized the urban transportation network, reduced the physical distance and commuting costs among enterprises, and profoundly impacted the exchange and collaboration between companies and universities/research institutions. According to social network theory and transaction cost theory (Rodan, 2010), improved transportation has lowered search and information costs between enterprises and universities, enhanced interaction frequency, and facilitated the establishment of collaborative ties for scientific research and innovation between companies and academic institutions (Lorincová et al., 2022). The convenience of the subway promotes information exchange and resource sharing between enterprises and universities, reinforcing the synergistic innovation effect. Frequent interactions encourage enterprises to seek cooperation with universities/research institutions, establish trust relationships, and form shared values and behavioral norms, thereby fostering the development and strengthening of a corporate culture of collaboration. This culture emphasizes team spirit, open sharing, and collaborative innovation, encouraging internal cross-departmental and external cross-sector collaboration, and promoting deep integration between enterprises and innovation sources. From the perspective of the internal effects of a collaborative culture on corporate innovation, it not only establishes information-sharing mechanisms within the enterprise but also unites employees and enhances the depth of R&D team collaboration, enabling each researcher to leverage their strengths within the team (Fiordelisi and Ricci, 2014). From the external effects, the guidance of a collaborative culture makes enterprises more inclined to establish long-term and stable cooperative relationships with universities, research institutions, and other innovation entities to jointly conduct technology research and patent applications. Enterprises with a collaborative culture are also better at seizing R&D cooperation opportunities with customers, suppliers, and research institutions, and can more effectively integrate each other's advantageous resources to achieve complementary advantages, resource sharing, and improve R&D success rates (Pan et al., 2019). Therefore, the opening of the subway system, by promoting the formation and enhancement of a corporate culture of collaboration, stimulates the vitality of Industry-academia-research collaboration, increases the quantity and quality of patents resulting from such collaborations, and provides important support for promoting corporate technological innovation and industrial upgrading. Based on the aforementioned theoretical analysis, we propose Hypothesis 2.

Research hypothesis two: The opening of the subway system enhances the corporate culture of collaboration, thereby promoting an increase in the level of Industry-academia-research collaboration among enterprises.

The opening of the subway system enhances the operational efficiency of urban transportation infrastructure, reduces travel costs, and strengthens collaboration between enterprises and financial institutions, universities and research institutions, and government entities. This not only elevates the innovation level of enterprises but also attracts more highly educated talent to these enterprises. According to human capital theory, As a key factor of production, the flow decision of talents is influenced by the cost-benefit balance. Improvements in transportation infrastructure can lower the cost of talent mobility, making it easier for highly educated individuals to move and congregate between cities. Furthermore, enhancing transportation efficiency effectively reduces commuting time, thereby increasing individual happiness (Li et al., 2018), raising the average wages of employees, or lowering unemployment rates. Improvements in the work environment and urban landscape manifestly enhance the overall image of the city, guiding the employment preferences of highly educated talent and subsequently bolstering the city's appeal for such talent due to the subway system. However, whether the inflow of highly educated talent induced by the subway opening is conducive to elevating the level of Industry-academia-research collaboration remains to be seen. In cities with high levels of industrial agglomeration or greater population diversity, the movement and exchange of highly educated individuals across different regions and enterprises facilitate the dissemination and diffusion of knowledge, thereby promoting technological progress. In particular, highly educated human capital exerts a significant impact on regional innovation levels (Wang and Yuan, 2022). Based on the complementary theory of capital-skill, the behavior of enterprises to hire more highly skilled labor force is essentially to strengthen the synergistic effect with physical capital by optimizing the structure of human capital. When enterprises increase capital investment such as advanced equipment and digital platforms, the professional knowledge of highly skilled labor force can significantly improve the utilization efficiency of capital elements. This complementary mechanism not only directly improves the transformation efficiency of enterprise R&D investment, but also drives the innovation of production process through knowledge spillover effect, and finally realizes the dual improvement of technological innovation ability and total factor productivity. Correspondingly, highly educated talent serves as the core workforce in enterprise R&D departments, and their loss can disrupt long-term project capital investments (Shen, 2021). Moreover, recruiting a large number of high-level graduates from local universities can shorten the enterprise's innovation cycle and mitigate the uncertainty of innovation outcomes (Wang and Yuan, 2022). Therefore, highly educated talent is crucial for enterprise Industry-academia-research collaboration. Based on the aforementioned theoretical analysis, we propose Hypothesis 3.

Research hypothesis three: The opening of the subway system increases the proportion of highly educated talent in enterprises, thereby promoting an elevation in the level of Industry-academia-research collaboration.

The opening of the subway system exerts a significant positive influence on enhancing the level of enterprise digital transformation, a viewpoint grounded in the widely recognized understanding within academic circles that improvements in transportation infrastructure boost the efficiency of corporate economic and innovation activities. As the density of subway lines increases, the extent of enterprise digital transformation rises accordingly, particularly for businesses located near city centers and within high-tech industries. Enterprise digital transformation necessitates substantial short-term investments and faces challenges such as considerable difficulty, lengthy duration, and high opportunity costs (Chen and Srinivasan, 2024). The subway reshapes urban spatial structures, enhances accessibility between regions, accelerates the speed of information dissemination, reduces information search and monitoring costs, and makes it easier for investors and high-tech talent to obtain company “soft information,” thereby bolstering investment willingness (Zhang et al., 2020) and promoting the inflow of human capital, providing ample financial and human capital support for enterprise digital transformation. According to neoclassical economic growth theory, which emphasizes the role of transportation facilities in driving economic growth, the opening of the subway offers enterprises a more convenient and efficient transportation environment. The optimization of this environment enables enterprises to more easily acquire and integrate information, thereby propelling them toward digital transformation. Furthermore, digital transformation is a crucial pathway for enterprise high-quality development, laying the foundation for an increase in the number of Industry-academia-research collaboration patents by enhancing data processing capabilities, optimizing business processes, and improving decision-making efficiency (Tang et al., 2023). Through digital means, enterprises can more readily share technical knowledge with universities, increasing the depth and breadth of their external knowledge search, thereby enhancing the outcomes and process performance of collaborative innovation (Li et al., 2024). Additionally, digital transformation assists enterprises in precisely grasping market demand, adjusting management behaviors and strategic decisions, promoting technological research and development, and improving patent protection systems, thereby facilitating Industry-academia-research collaboration (Yu et al., 2023). Consequently, by elevating the level of enterprise digital transformation, the opening of the subway indirectly promotes an increase in the number of Industry-academia-research collaboration patents, injecting new vitality into enterprise innovation development and industrial upgrading. Based on the aforementioned theoretical analysis, we propose Hypothesis 4.

Research hypothesis four: The opening of the subway enhances the level of enterprise Industry-academia-research collaboration by elevating the degree of enterprise digital transformation.

## 4 Research design

### 4.1 Data

This paper selects A-share listed companies from 2010 to 2022 as the research subjects, employing macro-micro matched data at the “city-enterprise” level to examine the impact of subway opening on enterprise Industry-academia-research collaboration. The selection of the sample period is primarily based on the following considerations: (1) To further improve and standardize the patent system, China initiated the revision of the “Patent Law of the People's Republic of China” in 2008, raising the standards for granting invention patents. This may have a certain impact on patent grants related to enterprise Industry-academia-research collaboration. In light of the time required for the new law to take effect and the availability of data, this paper designates 2010 as the starting year for the sample data. (2) Given the availability of regression panel data and the symmetry of the data sample time interval, this paper selects 2022 as the ending year for the sample data. To ensure data quality, the initial sample is processed as follows: (1) Financial listed companies are excluded; (2) ST, ST^*^, and PT enterprises within the sample interval are excluded; (3) Enterprises with missing sample data are excluded; (4) Winsorization at the 1% and 99% quantiles is applied to all continuous variables to mitigate the interference of outliers. The city-level data used in this paper is sourced from the “China City Statistical Yearbook,” subway opening data is obtained from the annual statistical reports of the China Association of Metros and government official websites, enterprise Industry-academia-research collaboration level data is derived from the patent retrieval system of the National Intellectual Property Administration (CNIPA), and other financial data of enterprises is sourced from the CSMAR database and the China Research Data Service Platform (CNRDS). Data processing and analysis are conducted using Stata 18.

### 4.2 Variable

#### 4.2.1 Explained variable

Industry-academia-research collaboration essentially refers to the concerted efforts among enterprises, research institutes, and higher education institutions, aiming to facilitate the effective transformation and application of scientific and technological achievements by integrating various production factors necessary for technological innovation. The method of measuring enterprise innovation capability through patent application volume has been widely adopted (Yin et al., 2022). Following the mainstream approach (Yang et al., 2022), this paper collects and organizes the actual patent application volume of Industry-academia-research collaboration for listed companies in the current period from the patent retrieval system of the National Intellectual Property Administration, using it as the dependent variable. Due to the significant disparities in the number of invention patent applications among listed companies, to mitigate the degree of right skewness in the data, this paper applies a logarithmic transformation (after adding 1) to the number of Industry-academia-research collaboration patent applications for enterprises in the given year.

Table 1 presents the statistics and comparisons of all variables between the experimental and control groups. It reveals that the experimental group significantly outperforms the control group in terms of the explained variable, indicating that industry-university-research collaboration in cities with subway systems is markedly superior to that in cities without such infrastructure, which foreshadows the baseline regression results. Although certain discrepancies exist among the control variables, they do not undermine the baseline regression findings. Furthermore, in the placebo test, this study employs PSM-DID to address the differences in control variables between the experimental and control groups, thereby ensuring the robustness of the fundamental conclusions.

**Table 1:** Inter-group differences.

Variable	G1(0)	Mean1	G2(1)	Mean2	MeanDiff
IAR	8,606	0.07	12,887	0.14	−0.07^***^
Size	8,606	22.03	12,887	22.07	−0.04^**^
Lev	8,606	0.41	12,887	0.41	0
ROE	8,606	0.04	12,887	0.04	0
Indep	8,606	2.13	12,887	2.12	0.02^***^
Board	8,606	0.37	12,887	0.38	−0.00^***^
Dual	8,606	0.26	12,887	0.3	−0.04^***^
Age	8,606	2.22	12,887	2.16	0.06^***^
Mshare	8,606	0.11	12,887	0.14	−0.03^***^
GDP	8,606	6.6	12,887	11.52	−4.92^***^
Indratio	8,606	48.45	12,887	40.14	8.30^***^
Finauto	8,606	0.56	12,887	0.81	−0.25^***^
Sci	8,606	2.48	12,887	4.34	−1.86^***^
Edu	8,606	0.19	12,887	0.16	0.03^***^
Findeve	8,606	0.02	12,887	0.05	−0.02^***^

^**^mean significant at the 5% level and

^***^mean significant at the 1% level.

#### 4.2.2 Core explanatory variable

The variable represents whether a city has opened a subway system in the given year, constructed by the interaction between the subway opening dummy variable (*Treatment*) and the policy implementation time dummy variable (*After*). As analyzed in the background, intra-city transportation infrastructure brings convenience to people's travel, enhances transportation accessibility and travel efficiency, and particularly, subways can significantly reduce the communication costs for researchers. Following the approach adopted in existing literature (Zheng et al., 2019), this paper uses the opening of a subway in a city as the criterion for dividing the treatment and control groups. If a city has opened a subway, *Treatment* is assigned a value of 1; otherwise, it is 0. *After* is a time dummy variable, which takes a value of 1 if the sample period is in the year of or after the subway opening, and 0 otherwise.

**Figure 1:**
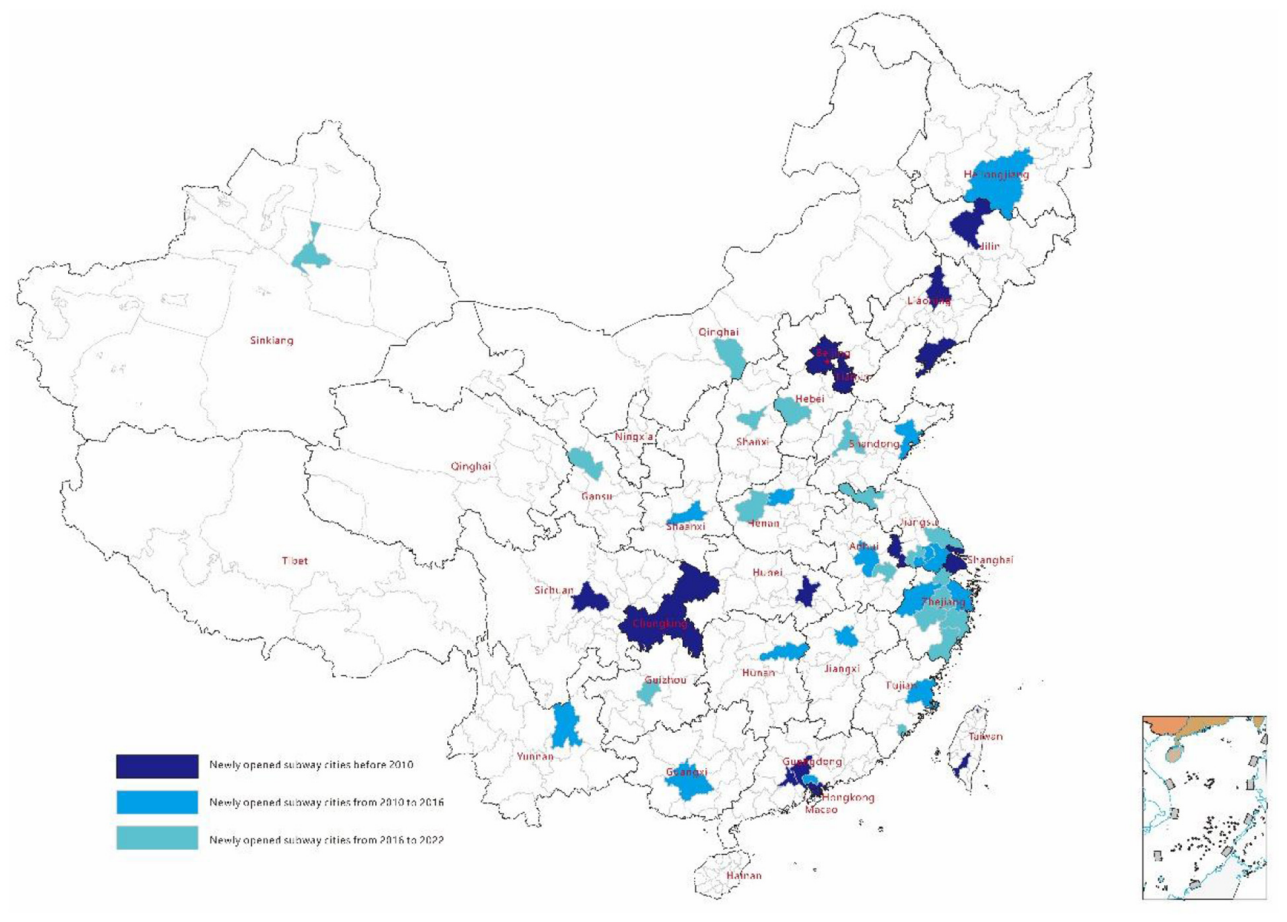
Chronological map of metropolitan subway system inaugurations.

This is a histogram of subway network expansion with time. Before 2010, there were fewer cities that opened subways, and the number of cities that opened subways increased year by year in the following years, which is why this study uses multi-time double difference to measure (Figure 1).

#### 4.2.3 Control variable

In order to account for potential influences of variations in enterprise nature and city resources on the level of Industry-academia-research cooperation, this paper adopts the methodology from existing literature (Zhong, 2018) and selects control variables from both enterprise and city characteristics. At the enterprise level, the control variables include: enterprise size (*Size*), debt-to-asset ratio (*Lev*), return on equity (*ROE*), proportion of independent directors (*Indep*), number of board members (*Board*), duality of CEO and chairman (*Dual*), years since listing (*Age*), and management shareholding ratio (*Mshare*). At the city level, the control variables encompass per capita GDP (*GDP*), proportion of the secondary industry (*Indratio*), fiscal autonomy (*Finauto*), intensity of scientific and technological investment (*Sci*), intensity of educational expenditure (*Edu*), and financial development indicator (*Findeve*).Descriptions of these variables are provided in Table 2. Table 2 shows all variables and their definitions.

**Table 2:** Variable description.

Variable category	Symbol	Variable name	Explanation
	(1)	(2)	(3)
Explained variable	Green	Industry-university-research institute cooperation	ln (1 + Patent applications of industry-university-research institute cooperation in the current year)
Explanatory variable	Treatment	Dummy variable for subway opening	Treatment takes 1 if the city has a subway opened, otherwise 0
	After	Dummy variable for policy implementation	After takes 1 if it is the opening year and later, otherwise 0
Firm-level control variables	Size	Enterprise scale	ln (Total assets at the end of the year)
	Lev	Asset-liability ratio	Total liabilities at the end of the year/total assets at the end of the year
	ROE	Return on net assets	Net profit/average balance of shareholders' equity
	Indep	Proportion of independent director	Number of independent directors/number of directors
	Board	Number of directors	ln (Number of directors)
	Dual	Dual-role of CEO and chairman	Dual takes 1 if the chairman and the general manager are the same person, otherwise 0
	Age	Years since listing	ln (Current year – Year of listing + 1)
	Mshare	Proportion of management shareholding	Number of shares held by directors, supervisors and senior management/total number of shares
City-level control variables	GDP	Per capita GDP of the city	GDP/total population
	Indratio	Proportion of secondary industry	Output value of secondary industry/GDP
	Finauto	Fiscal autonomy	Local general public budget expenditure/local general public budget revenue
	Sci	Intensity of science and technology investment	Science and technology expenditure/local general public budget expenditure
	Edu	Intensity of education expenditure	Education expenditure/local general public budget expenditure
	Findeve	Financial development index	Total deposits and loans of financial institutions each year as a proportion of GDP

### 4.3 Identification

To investigate the causal effect of subway openings on Industry-academia-research cooperation among enterprises, this paper employs the opening of subways in various cities across different years as an exogenous shock. The Difference-in-Differences method can effectively control individual heterogeneity and regional characteristics that do not change over time by constructing an experimental group (policy implementation area) and a control group (policy non implementation area), and comparing the differences between the two groups before and after the policy, reducing endogeneity problems caused by omitted variables. Compared with traditional regression analysis, its conclusions have stronger causal inference ability, providing more reliable evidence support for policy evaluation and theoretical verification. Based on whether a city has opened a subway, the study divides the sample into an experimental group and a control group, and utilizes the Difference-in-Differences method to establish the following benchmark regression model:


$$\begin{array}{l} IAR_{i,t}=\beta_{0}+\beta_{1}Treatment_{i}\times After_{t}+\gamma Controls_{i,t} \end{array}$$ (1)

$$\begin{array}{l} \;+\delta_{i}+\theta_{t}+ind_{i}+\varphi_{i,t} \end{array}$$

Wherein, subscript i and t represent the enterprise and year, respectively; the dependent variable IAR_i, t denotes the level of Industry-academia-research cooperation for enterprise*i* in year t; Treatment_i_ is a dummy variable for subway opening (taking a value of 1 if the city has opened a subway, otherwise 0), and After_t_ is a policy implementation dummy variable (taking a value of 1 for the year of opening and subsequent years, otherwise 0). Controls_i, t_ represent control variables encompassing both enterprise-level and city-level factors, δ_*i*_ represents firm fixed effects, θ_*t*_ represents time fixed effects and φ_*i, t*_ represents the random error term.

The urban subway construction in China has obvious policy externalities, and the planning approval is strictly controlled by the National Development and Reform Commission, not driven by the city's own economy or innovation ability. Its construction funds are mainly financial investment, and the project promotes the related government performance evaluation and people's livelihood needs, reflecting the strategic orientation's regulation of market factors. The planning period is 5–8 years. After the opening sequence is incorporated into the overall urban planning, it is less adjusted due to short-term economic fluctuations, and it is independent of the current development indicators, with strong stability. In the research design, this study controls the inherent characteristics of the city and the macro-cycle influence through a multi-dimensional fixed effect model, so as to identify the differences of policy shocks in different periods of the same city. At the same time, the endogenous interference in the administrative decision-making of subway construction is stripped in the model, and its core driving factors are determined by the institutional framework at the super-city level, which is the endogenous result of the current economic and innovation capacity of non-cities. This externality definition conforms to the “policy first” characteristics of China's infrastructure, and provides a reasonable premise for causal inference.

## 5 Empirical results

### 5.1 Descriptive statistics

Table 3 presents the descriptive statistics for the key variables. According to this table, the mean of patent applications for Industry-academia-research cooperation among enterprises is 0.110, with a median of 0.0770, a standard deviation of 0.356, a minimum value of 0, and a maximum value of 5.385. These figures indicate considerable variation in the level of Industry-academia-research cooperation among different enterprises and suggest that the overall level of cooperation is relatively low, warranting further enhancement. The mean of the subway opening dummy variable, *Treatment* is 0.600, indicating that 60.0% of enterprises are located in cities with subway systems, which aligns with the requirements of the Difference-in-Differences method for the size of the treatment group. The mean of the policy implementation dummy variable, *After* is 0.476, approaching 1/2, suggesting that the sample sizes of enterprises located in cities with and without subway systems before and after the opening are roughly equivalent, ensuring good symmetry of the sample interval in the temporal dimension. The descriptive statistics for the remaining variables are generally consistent with previous research and do not exhibit significant skewness, fulfilling the basic requirements of the Difference-in-Differences method for data samples.

**Table 3:** Descriptive statistics.

Variables	Observations	Mean	Median	Standard deviation	Minimum	Maximum
	**(1)**	**(2)**	**(3)**	**(4)**	**(5)**	**(6)**
IAR	21,493	0.110	0.077	0.356	0.000	5.385
Treatment	21,493	0.600	1.000	0.490	0.000	1.000
After	21,493	0.476	0.000	0.499	0.000	1.000
Size	21,493	22.058	21.913	1.211	17.641	27.621
Lev	21,493	0.414	0.409	0.199	0.007	2.128
ROE	21,493	0.039	0.038	0.077	−1.146	1.285
Indep	21,493	37.429	33.330	5.558	0.000	80.000
Board	21,493	2.125	2.197	0.193	0.693	2.890
Dual	21,493	0.280	0.000	0.449	0.000	1.000
Age	21,493	2.187	2.303	0.735	0.000	3.497
Mshare	21,493	13.063	0.616	19.265	0.000	89.725
GDP	21,493	9.549	8.868	4.718	1.808	20.349
Indratio	21,493	43.469	44.880	10.401	15.878	63.910
Finauto	21,493	0.714	0.759	0.218	0.046	1.541
Sci	21,493	0.039	0.035	0.025	0.003	0.130
Edu	21,493	0.171	0.165	0.036	0.098	0.260
Findeve	21,493	3.597	3.320	1.587	1.207	7.506

### 5.2 Benchmark regression

To ensure the robustness of the empirical results, this study incrementally incorporates control variables at both the firm and city levels into Model (1) for estimation. Table 4 presents the regression results regarding the impact of subway access on the level of Industry-academia-research cooperation among enterprises. Specifically, Column (1) excludes control variables and reveals that the regression coefficient for whether a subway was opened in the city in the current year (*Treatment*_*i*_×*After*_*t*_) is 0.046, passing the significance test at the 1% level. This indicates that subway access has a significant positive effect on Industry-academia-research cooperation among enterprises. Furthermore, Column (2) presents the regression results after incorporating firm-level control variables based on Column (1). Lastly, Column (3) presents the regression results after incorporating city-level control variables based on Column (2). The results demonstrate that the regression coefficients of the core explanatory variable are consistently significant and positive. The regression results in Column (3) indicate that subway access promotes an increase of 4.4% in the level of industrial cooperation among enterprises at the 1% confidence level. Before and after the inclusion of control variables, the estimated coefficient of the explanatory variable *Treatment*_*i*_×*After*_*t*_ decreases slightly, suggesting that the selection of control variables is reasonable.

**Table 4:** Benchmark regression.

Variables	IAR	IAR	IAR
	**(1)**	**(2)**	**(3)**
Treatment × After	0.046^***^	0.047^***^	0.044^***^
	(0.014)	(0.014)	(0.014)
Size		0.034^***^	0.034^***^
		(0.007)	(0.007)
Lev		−0.013	−0.014
		(0.030)	(0.030)
ROE		−0.015	−0.013
		(0.032)	(0.032)
Indep		−0.015	−0.016
		(0.027)	(0.027)
Board		0.023	0.028
		(0.077)	(0.078)
Dual		0.012	0.012
		(0.008)	(0.008)
Age		−0.033^**^	−0.034^**^
		(0.015)	(0.015)
Mshare		0.035	0.035
		(0.030)	(0.030)
GDP			0.002
			(0.002)
Indratio			−0.001
			(0.001)
Finauto			−0.026
			(0.047)
Sci			0.003
			(0.008)
Edu			0.258
			(0.159)
Findeve			0.433
			(0.319)
Constant	0.088^***^	−0.579^***^	−0.587^***^
	(0.007)	(0.148)	(0.163)
Year-FE	Yes	Yes	Yes
Firm-FE	Yes	Yes	Yes
Adj.*R*^2^	0.448	0.450	0.451
Observations	21,493	21,493	21,493

The values in parentheses represent robust standard errors for clustering at the city level. ^***^, ^**^, and ^*^ indicate significance levels of 1%, 5%, and 10%, respectively. Column (1) presents the regression results for a single variable, column (2) includes the regression results with the inclusion of the enterprise level control variable, and column (3) shows the regression results with all control variables included. All regressions control for time fixed effects, firm fixed effects, and city fixed effects. ^*^means significant at the 10% level.

### 5.3 Robustness test

#### 5.3.1 Parallel trend test

The parallel trends assumption is a prerequisite for employing the Difference-in-Differences model. It posits that the dependent variable in both the treatment and control groups would exhibit similar trends in the absence of the policy. Failure to satisfy this assumption suggests that the policy outcomes reported in this study may be attributable to other omitted variables. This study utilizes a sample of China's A-share listed companies from 2010 to 2022. During this period, subway systems were inaugurated in various cities nearly every year, resulting in potential event periods including {−12, −11, −10, −9, −8, −7, −6, −5, −4, −3, −2, −1, 0, 2, 3, 4, 5, 6, 7, 8, 9, 10, 11, 12…}, where -i denotes the i-th period before policy implementation and i denotes the i-th period after. To ensure comparable sample sizes across periods, the pre-implementation periods from 12 to 3 were consolidated into pre-period 3, the current period remained period 0, and post-implementation periods from 3 to 12 were merged into post-period 3. Consequently, the “event” periods were defined as {−3, −2, −1, 0, 2, 3} relative to policy implementation, with pre-period 3 designated as the benchmark. The specific econometric model is as follows:


$$\begin{array}{l} IAR_{i,t}=\alpha +\beta_{n}\overset{3}{\underset{n=-2}{\sum }}Treatment_{i}\times After_{t}^{n} \end{array}$$ (2)

$$\begin{array}{l} \;+\gamma Controls_{i,t}+\delta_{i}+\theta_{t}+ind_{i}+\varphi_{i,t} \end{array}$$

**Figure 2:**
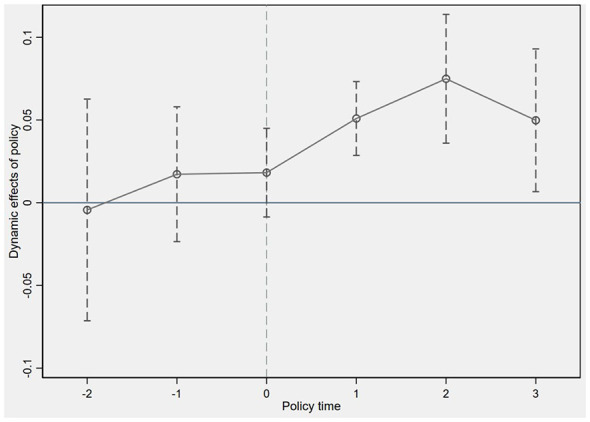
Parallel trend test 1. In the figure, the short dashed lines indicate confidence intervals, the hollow dots represent estimated coefficients, the horizontal coordinates indicate the years before and after policy implementation, while the vertical coordinates indicate coefficient values. We use the year of the policy as the base year and have labeled it in the figure by the long dotted line.

Wherein, the dependent variable remains the number of patent applications for Industry-academia-research cooperation among enterprises, and β_*n*_ reflects the effect before and after policy implementation. If the coefficient of β_*n*_ is zero prior to policy implementation, i.e., when n is less than 0, this indicates that the parallel trends assumption is satisfied. The parallel trends assumption in this study is illustrated in Figure 2, where the insignificant regression coefficients prior to subway opening suggest no significant differences between the treatment and control groups before implementation. Conversely, the coefficients for the year of subway opening and subsequent years increase significantly, indicating that the treatment group has made notable progress in Industry-academia-research cooperation. This result validates the effectiveness of the baseline regression results and passes the parallel trends test.

#### 5.3.2 Placebo test

**Figure 3:**
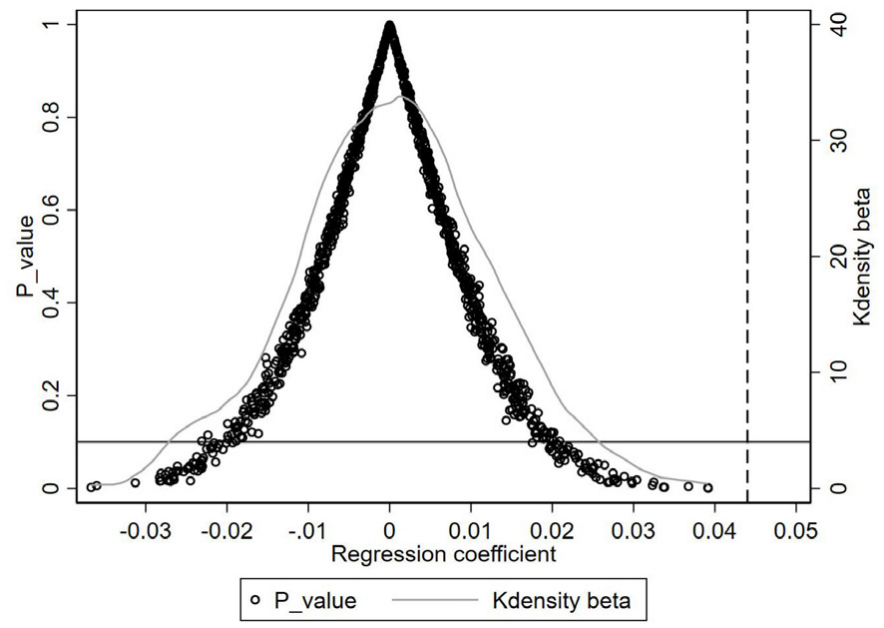
Placebo test. In the figure, solid circles represent the regression outcomes for each simulated policy variable. The horizontal axis denotes the regression coefficient of each simulated policy variable, the left vertical axis signifies the *P*-value, and the right vertical axis indicates the kernel density.

In order to exclude the influence of other unobservable confounding factors on the regression results and obtain more reliable causal effects, we follow the mainstream practices in existing literature (Fang et al., 2014) to conduct a test. Specifically, a counterfactual test is performed by randomly assigning the subway opening policy to create “pseudo-treatment” and “pseudo-control” groups, obtaining pseudo-policy interaction terms, and re-running the regression using Model (1). Through 1,000 random samples, 1,000 “fake” estimated coefficients are obtained and plotted in a kernel density graph, as shown in Figure 3. Under random assignment, the estimated coefficients of the explanatory variable (*Treatment*_*i*_×*After*_*t*_) are symmetrically distributed around zero, and the baseline regression result of 0.044 is significantly different from the placebo test coefficients. Therefore, it can be concluded that the baseline regression results in this study are not driven by chance factors, further supporting the validity of the baseline regression results.

Robustness test two: To further validate the policy effect, we conducted a counterfactual test by advancing the implementation of the subway opening policy by 2 years. Column (1) in Table 5 shows that the regression coefficient of the explanatory variable (*Treatment*_*i*_×*After*_*t*_) is positive but insignificant, thereby confirming that the subway opening can promote Industry-academia-research cooperation among enterprises.

**Table 5:** Placebo test.

Variables	IAR	IAR_sf	IAR	IAR	Intensity	Breadth	IAR
	**(1)**	**(2)**	**(3)**	**(4)**	**(5)**	**(6)**	**(7)**
Treatment × (After-2)	0.008						
	(0.016)						
Treatment × After		0.008^***^	0.045^***^	0.045^***^	0.037^***^	0.008^***^	0.044^***^
		(0.003)	(0.015)	(0.014)	(0.009)	(0.003)	(0.013)
High-tech enterprise			−0.000				
			(0.013)				
R&D				−0.038^**^			
				(0.018)			
Controls	Yes	Yes	Yes	Yes	Yes	Yes	Yes
Year-FE	Yes	Yes	Yes	Yes	Yes	Yes	Yes
Firm-FE	Yes	Yes	Yes	Yes	Yes	Yes	Yes
City-FE							Yes
Adj.*R*^2^	0.452	0.253	0.452	0.453	0.274	0.249	0.451
Observations	21,493	21,493	21,493	21,493	21,493	21,493	21,493

The values in parentheses represent robust standard errors for clustering at the city level. ^***^, ^**^, and ^*^ indicate significance levels of 1%, 5%, and 10%, respectively. All regressions control for time fixed effects, firm fixed effects, and city fixed effects. ^*^means significant at the 10% level.

Robustness test three: We replaced the dependent variable with a dummy variable indicating whether the enterprise continued its Industry-academia-research cooperation and re-ran the regression. Column (2) in Table 5 reveals that the regression coefficient of the explanatory variable (*Treatment*_*i*_×*After*_*t*_) is significantly positive at the 1% level, further indicating that the subway opening can effectively enhance the level of Industry-academia-research cooperation among enterprises.

Robustness test four: To control for the influence of enterprise characteristics and exclude the impact of technological innovation attributes, we included a dummy variable indicating whether the enterprise is a high-tech enterprise in the regression analysis based on Model (1). Column (3) in Table 5 demonstrates that the regression coefficient of the explanatory variable (*Treatment*_*i*_×*After*_*t*_) is significantly positive at the 1% level, further proving the robustness of the baseline regression results.

Robustness test five: The research and development cost plus deduction policy directly reduces enterprise research and development costs and enhances their enthusiasm for innovation investment. To accurately identify the independent effect of the subway opening and exclude potential confounding effects from other policies implemented during the same period, we controlled for the interference of the research and development cost plus deduction policy. Column (4) in Table 5 shows that after controlling for the research and development cost policy factor, the regression coefficient of the explanatory variable (*Treatment*_*i*_×*After*_*t*_) remains significantly positive at the 1% level, validating the baseline regression results.

Robustness test six: In order to study the intensity and breadth of the cooperation effect of subway on Industry-University-Research, we reconstructed the variables in model (1). We use the ratio of the number of enterprises' cooperation in Industry-University-Research to the total number of cooperative patents to show the intensity, and explore its breadth through whether to cooperate in Industry-University-Research. Columns (5) and (6) in Table 5 show that the regression coefficient of explanatory variables (*Treatment*_*i*_×*After*_*t*_) is significantly positive at 1% level, validating the baseline regression results.

Robustness test seven: In order to further test the influence of the change of standard error clustering level on the policy effect, the standard error clustering is classified to the regional level, and the empirical results are shown in the column (7) of Table 5. Compared with the benchmark estimation results, the change of standard error clustering level has not affected the size and significance of the estimated value of policy virtual variable coefficient. Therefore, the conclusion of this paper is credible regardless of whether the standard is wrongly clustered to the enterprise level or the city level.

### 5.4 Mediation mechanism test

Based on the theoretical analysis presented earlier, the positive incentive effect of subway opening on Industry-academia-research cooperation among enterprises is primarily achieved through three pathways: enhancing the enterprise's cooperative culture, promoting human capital upgrading, and increasing the enterprise's digitalization level. Drawing inspiration from Jiang's (2022) research, this paper constructs Model (3) to test these underlying mechanisms. Specifically, Model (3) replaces the dependent variable (*IAR*_*i, t*_) in Model (1) with the mediator variable (Mediater_i, t_), while all other variables remain unchanged.


$$\begin{array}{l} Mediater_{i,t}=\beta_{0}+\beta_{1}Treatment_{i}\times After_{t} \end{array}$$ (3)

$$\begin{array}{l} \;+\gamma Controls_{i,t}+\delta_{i}+\theta_{t}+ind_{i}+\varphi_{i,t} \end{array}$$

Drawing upon existing literature (Loughran and McDonald, 2013), this paper employs keyword frequency analysis to ascertain whether a corporate culture embraces collaboration. After establishing a lexicon pertinent to collaborative culture (Fiordelisi and Ricci, 2014), we utilize textual analysis to count occurrences of collaboration-related terms and their synonyms in the board report section of annual reports, normalizing this count by the total number of words in that section. A higher value of this indicator signifies a stronger corporate culture of collaboration. Column (1) of Table 5 presents the test results for the impact of subway opening on corporate collaboration culture, revealing that the regression coefficient for the explanatory variable (*Treatment*_*i*_×*After*_*t*_) is significantly positive at the 1% level, indicating that subway opening effectively enhances corporate collaboration culture. Based on this analysis, subway opening positively influences Industry-academia-research cooperation among enterprises by fostering a corporate culture of collaboration, thereby confirming Research Hypothesis 2. Subway opening reduces communication and transaction costs among enterprises and between enterprises and universities, promotes increased interaction frequency in collaborative endeavors, fosters long-term stable partnerships, deepens the formation of a collaborative culture, stimulates the vitality of Industry-academia-research cooperation, and enhances the quantity and quality of Industry-academia-research patents, providing crucial support for driving technological innovation and industrial upgrading among enterprises.

This paper measures the level of corporate human capital (Kafouros et al., 2015) using the proportion of employees with graduate degrees or higher. Column (2) of Table 5 shows the test results for the impact of subway opening on corporate human capital structure, indicating that the regression coefficient for the explanatory variable (*Treatment*_*i*_×*After*_*t*_) is significantly positive at the 1% level, demonstrating that subway opening significantly increases the proportion of employees with graduate degrees or higher, thereby promoting the optimization and upgrading of corporate human capital structure. Taken together, these findings validate Research Hypothesis 3, which posits that subway opening can facilitate cooperation among enterprises and various parties by promoting human capital upgrading, attracting highly educated talent, reducing talent mobility costs, enhancing city image and talent attractiveness, and ultimately strengthening the level of Industry-academia-research cooperation among enterprises, fostering technological innovation, and attracting more venture capital.

To quantify the level of corporate digital transformation, this study adopts a prevalent methodology by analyzing the frequency of digital-related terms in corporate annual reports and then taking the logarithm of this frequency plus one, serving as an indicator to measure the extent of corporate digital transformation (Wu et al., 2021). Column (3) of Table 6 presents the test results for the impact of subway opening on corporate digitization, revealing that the regression coefficients for the explanatory variable (*Treatment*_*i*_×*After*_*t*_) are significantly positive at the 5% level, indicating that subway opening notably enhances corporate digitization and promotes the optimization and upgrading of corporate human capital structure. In summary, these findings validate Research Hypothesis 4, which posits that subway opening optimizes the process of information acquisition and integration within enterprises, enhances the efficiency of information gathering, processing, and decision-making communication, thereby serving as a crucial pathway to drive corporate digital transformation and high-quality development. Moreover, by facilitating the sharing of technological knowledge, grasping market demand dynamics, and facilitating management adjustments and technological innovation, subway opening injects new vitality into the enhancement of Industry-academia-research cooperation among enterprises.

**Table 6:** Mediating mechanism test.

Variables	Cooperative culture	Proportion of postgraduate and above	Digital transformation
	**(1)**	**(2)**	**(3)**
Treatment × After	1.312^***^	0.538^***^	0.083^**^
	(0.501)	(0.141)	(0.041)
Year-FE	Yes	Yes	Yes
Firm-FE	Yes	Yes	Yes
Adj.*R*^2^	0.572	0.905	0.737
Observations	21,270	13,284	21,493

The values in parentheses represent robust standard errors for clustering at the city level. ^***^, ^**^, and ^*^ indicate significance levels of 1%, 5%, and 10%, respectively.

All regressions control for time fixed effects, firm fixed effects, and city fixed effects. ^*^means significant at the 10% level.

### 5.5 Heterogeneity discussion

This paper further examines the differences in the impact of subway opening on Industry-academia-research cooperation among enterprises with varying ownership structures, technological capabilities, and sizes, clarifying the mechanism through which heterogeneous factors exert their influence. The findings provide empirical evidence and targeted decision-making support for governments to further optimize subway construction and enhance the level of Industry-academia-research cooperation among enterprises.

#### 5.5.1 Heterogeneity in ownership structure

To examine the differential impact of subway opening on Industry-academia-research cooperation among enterprises with varying ownership structures, this study divides the data samples into state-owned enterprises and non-state-owned enterprises for grouped regression analysis. The regression results are presented in Columns (1) and (2) of Table 6, with Column (1) showing the regression results for state-owned enterprises and Column (2) for non-state-owned enterprises. According to the regression results, the estimated coefficient for state-owned enterprises is not significant, whereas the estimated coefficient for non-state-owned enterprises is significantly positive at the 5% confidence level, indicating that subway opening significantly enhances the level of Industry-academia-research cooperation among non-state-owned enterprises. When confronted with policy shocks, enterprises with different ownership structures may exhibit differentiated response speeds. According to the principal-agent theory in institutional economics, specifically, when making business decisions, state-owned enterprises consider factors beyond mere economic profit maximization, also taking into account multiple non-economic objectives such as social stability and national strategy implementation. This diversified goal system results in a certain time lag effect when state-owned enterprises respond to adjustments such as subway opening policies. Compared with the enterprise theory of neoclassical economics, as market entities independent of the government, non-state-owned enterprises formulate their business decisions highly dependent on market signals and changes in economic benefits, demonstrating a keener perception of changes in the external environment. In the specific context of subway opening policies, According to the theory of environmental perception in organizational behavior, non-state-owned enterprises are more likely to swiftly capture shifts in policy orientation, such as the increase in commercial real estate value and consumption potential along subway lines due to increased foot traffic. Based on these insights, non-state-owned enterprises may promptly adjust their business layouts to optimize resource allocation.

#### 5.5.2 Heterogeneity in industry attributes

To examine the differential impact of subway opening on Industry-academia-research cooperation among enterprises with varying industry attributes, this study divides the data samples into high-tech enterprises and non-high-tech enterprises for grouped regression analysis. The regression results are presented in Columns (3) and (4) of Table 6, with Column (3) showing the regression results for high-tech enterprises and Column (4) for non-high-tech enterprises. According to the regression results, the estimated coefficient for non-high-tech enterprises is not significant, whereas the estimated coefficient for high-tech enterprises is significantly positive, indicating that subway opening has a significant promotional effect on Industry-academia-research cooperation among high-tech enterprises. There exists a notable difference in the response of high-tech vs. non-high-tech enterprises to subway opening policies. High-tech enterprises, which typically rely on the rapid flow of high-end talent and innovative resources, tend to be more sensitive to transportation convenience. The opening of subways not only significantly reduces employee travel costs and enhances work efficiency but also strengthens connections between enterprises and their upstream and downstream partners, as well as research institutions and universities, facilitating rapid exchange of knowledge and technology. Therefore, high-tech enterprises are more likely to actively respond to subway opening policies by locating along subway lines or optimizing their office layouts to maximize the utilization of this transportation advantage. They may even further increase R&D investment and talent recruitment efforts to accelerate the innovation process within the enterprise. In contrast, although non-high-tech enterprises will also benefit from subway opening, their response speed and depth in leveraging this policy dividend for strategic adjustments and innovation upgrades may be relatively limited.

#### 5.5.3 Heterogeneity in enterprise size

To examine the differential impact of subway opening on Industry-academia-research cooperation among enterprises of varying sizes, this study divides the data samples into large-scale enterprises and small-scale enterprises for grouped regression analysis. The regression results are presented in Columns (5) and (6) of Table 7, with Column (5) showing the regression results for large-scale enterprises and Column (6) for small-scale enterprises. According to the regression results, the estimated coefficient for large-scale enterprises is not significant, whereas the estimated coefficient for small-scale enterprises is significantly positive. This indicates that, although large-scale enterprises can also benefit from subway opening, small-scale enterprises tend to perform more impressively in promoting Industry-academia-research cooperation and accelerating technological innovation. Enterprises of different sizes exhibit distinct response patterns when faced with subway opening policies. Large-scale enterprises, with abundant resources, benefit from the convenience in logistics and personnel mobility brought about by subway opening, which helps them increase cooperation opportunities and consolidate their market position. However, small-scale enterprises often demonstrate more significant effects in promoting Industry-academia-research cooperation. Small-scale enterprises possess more flexible management structures and operational processes, enabling them to quickly adapt to market changes and seize new opportunities presented by subway opening policies. The opening of subways makes it easier for small-scale enterprises to establish connections with nearby research institutions and innovation centers, introducing new technologies and knowledge through Industry-academia-research cooperation to compensate for their deficiencies in research and innovation, thereby substantially enhancing their core competitiveness. Furthermore, small-scale enterprises have shorter decision-making chains and can rapidly convert cooperation achievements into actual productivity, allowing them to stand out in the fierce market competition.

**Table 7:** Heterogeneity analysis.

Variables	State-owned	Non-state-owned	High-tech	Non-high-tech	Large-scale	Small-scale
	**(1)**	**(2)**	**(3)**	**(4)**	**(5)**	**(6)**
Treatment × After	1.178	1.382^**^	1.753^***^	0.594	0.358	2.327^***^
	(0.864)	(0.649)	(0.591)	(0.918)	(0.723)	(0.680)
Year-FE	Yes	Yes	Yes	Yes	Yes	Yes
Firm-FE	Yes	Yes	Yes	Yes	Yes	Yes
Observations	6,709	14,075	12,383	8,745	10,794	10,476
Adj.*R*^2^	0.592	0.581	0.585	0.628	0.585	0.558

The values in parentheses represent robust standard errors for clustering at the city level. ^***^, ^**^, and ^*^ indicate significance levels of 1%, 5%, and 10%, respectively. ^*^means significant at the 10% level.

## 6 Research conclusions and policy recommendations

### 6.1 Research conclusions

Advancing Industry-academia-research innovation collaboration is a pivotal driver for promoting high-quality enterprise development and enhancing the overall national innovation efficiency. The question of how to strengthen Industry-academia-research collaboration among enterprises is a topic of mutual concern for policymakers and university in the new era. Currently, countries are actively developing urban transportation infrastructure, aiming to build and advance transportation powerhouses while further elevating the level of Industry-academia-research collaboration among enterprises. This paper takes Industry-academia-research collaboration patents as the entry point and employs a multi-period Difference-in-Differences model to conduct an empirical analysis of the relationship between subway opening and Industry-academia-research collaborative innovation, using listed enterprises in Shanghai and Shenzhen from 2011 to 2022 as the research sample. The research findings indicate that the opening of subways significantly enhances the level of Industry-academia-research collaborative innovation among enterprises located along the subway lines, resulting in an approximately 4% increase in the number of Industry-academia-research collaboration patent applications. These results remain valid after various robustness checks. An analysis of intermediary mechanisms reveals that the incentive effect of subway opening on Industry-academia-research collaboration among enterprises is primarily achieved through three pathways: strengthening the enterprise's collaborative culture, increasing the proportion of high-quality talent, and advancing the level of enterprise digital transformation. After further considering the heterogeneous impact of enterprise nature, the improved level of Industry-academia-research collaboration among enterprises resulting from the opening of urban subways is more pronounced for non-state-owned enterprises, high-tech enterprises, and smaller-scale enterprises. This study analyzes the promotional effect of subway opening on the level of Industry-academia-research collaboration among enterprises from multiple perspectives. Based on the triple helix theoretical framework, this study has achieved innovative expansion of the practical dimension, constructed an analytical framework integrating the perspective of spatial economics, and broke through the flat explanation of the cooperative relationship of innovative subjects in traditional theories, providing a more adaptable theoretical tool for understanding how infrastructure investment reshapes political Industry-University-Research. And this study provides robust support for policymakers to further comprehensively enhance the level of Industry-academia-research collaboration among enterprises and promote the development of transportation infrastructure such as subways.

### 6.2 Policy recommendations

From the perspective of international experience and development trend, the above conclusions can be combined with the adaptive adjustment of different institutional environments and development stages to provide a solution with both theoretical reference value and practical operation significance for promoting regional innovation synergy relying on rail transit infrastructure around the world, this paper proposes the following three feasible policy recommendations.

First, intensify subway construction efforts and enhance policy support for Industry-academia-research collaboration along subway lines. The government should prioritize not only the direct benefits of subway construction but also the resulting flow and agglomeration of innovation resources. By accelerating the development of the subway network and enhancing transportation efficiency, the government can ensure that residents‘ travel needs are met while facilitating enterprise innovation collaboration. Additionally, policy support should be increased for Industry-academia-research collaboration projects along subway lines, with route design incorporating more enterprises and universities to achieve rapid connectivity, accelerate information flow, and harness the knowledge spillover effect. The government may establish special funds to prioritize support for Industry-academia-research projects along subway lines, fostering the concentration of innovation resources, forming innovation clusters, enhancing enterprises' innovation capabilities and market competitiveness, and expanding the innovation-driven path for high-quality industrial development. To strengthen the innovation driven effect of industry university research cooperation projects along the subway line, policy incentives need to be formulated in a hierarchical and classified manner. Direct subsidies, tax exemptions, and PPP models are applicable to projects at different stages, each with its own advantages and disadvantages. Based on the characteristics of the project, which combines public attributes and market vitality, it is recommended to adopt a combination strategy of “PPP led, tax reduction supplemented, and precise direct subsidy assisted”: using PPP to attract social capital to build technology complexes, incentivizing enterprise research and development transformation with tax reduction, providing competitive direct subsidies to cutting-edge fields, and forming a multi-party collaborative innovation ecosystem.

Second, strengthen corporate collaboration culture, increase the proportion of high-quality talent in enterprises, accelerate digital transformation, and create an innovative development ecosystem for Industry-academia-research collaboration. According to mechanism analysis results, the opening of subways promotes Industry-academia-research collaboration among enterprises by strengthening their collaboration culture, increasing highly educated talent, and enhancing their digital capabilities. The government should encourage enterprises to strengthen their internal collaboration culture, provide financial support and incentive mechanisms, and share the costs and pressures of digital innovation, green innovation, and social responsibility fulfillment, thereby promoting enterprises to invest resources in innovation collaboration. Furthermore, the government can establish information-sharing platforms to enhance transparency, reduce information asymmetry, and provide efficient channels for cooperation and communication among innovation entities. The government should also optimize talent policies, support joint training of high-level talent between enterprises and universities, launch talent introduction programs, and enhance the attractiveness of regions along subway lines for talent. Additionally, the government should encourage enterprises to increase investments in digitization, provide financial subsidies and tax incentives, and promote the deep integration of digital technology and innovation collaboration projects, ensuring the technological advancement of Industry-academia-research collaboration among enterprises.

Third, improve the precision and effectiveness of policy support to maximize the Industry-academia-research promotion effect of subway opening policies. According to the heterogeneity analysis results in this paper, the opening of subways has a more significant effect on enhancing Industry-academia-research collaboration innovation among non-state-owned enterprises, high-tech enterprises, and small-scale enterprises. The country should emphasize the role of non-state-owned enterprises, high-tech enterprises, and small-scale enterprises as key players in Industry-academia-research collaboration and formulate differentiated policies. The government should broaden financing channels for non-state-owned enterprises, provide research and development tax relief for high-tech enterprises, and offer flexible cooperation platforms for small-scale enterprises. At the same time, state-owned enterprises should be encouraged to optimize resource integration and deepen Industry-academia-research collaboration. Policies should also focus on enterprises with room for technological improvement, leveraging the innovation spillover effects of digital enterprises to drive enterprises across industries and sizes, promote the transformation of scientific and technological achievements, and expand the application scenarios of Industry-academia-research collaboration.

By refining the targets of policy formulation, the government can tailor policies to meet the innovation needs of different types of enterprises. Driven by subway opening policies, the density and breadth of Industry-academia-research collaboration can be enhanced, improving the overall innovation capability of society and providing robust policy support for the sustainable development of the country.

### 6.3 Limitations

This study possesses certain limitations and offers suggestions for future research directions. Firstly, it exclusively focuses on publicly traded companies as the subjects of analysis, ensuring data availability and symmetry, yet neglects non-publicly traded companies, particularly the performance and contributions of small and medium-sized enterprises in Industry-academia-research collaborations. As significant forces in technological innovation, non-publicly traded companies' unique effects and innovative collaboration models influenced by the opening of subway lines have not been adequately explored, thereby limiting the universal applicability of the research conclusions. Secondly, while this study conducts an in-depth analysis using China as an example, there may be regional and cultural limitations in directly generalizing the conclusions to a global context. Moreover, the treatment group is defined as “the city where the subway is opened,” and the spatial heterogeneity of subway radiation is not considered. For example, the difference between the enterprises in the outer suburbs and the enterprises in the city center is not reflected, and the sensitivity analysis with the distance from the enterprise to the subway station as the treatment intensity variable is not discussed, which reduces the transparency of the results. Lastly, regarding the evaluation of Industry-academia-research collaboration outcomes, this study primarily relies on a single indicator of patent counts, supplemented by the output of research projects and initiatives. The number of patents alone fails to comprehensively reflect the quality, effectiveness, and long-term socioeconomic impacts of the collaborations. Furthermore, the contributions of Industry-academia-research collaborations in areas such as technology transfer, talent cultivation, and industrial upgrading have not been adequately quantified or deeply explored in this study, impacting the breadth of the research conclusions.

## Data Availability

Publicly available datasets were analyzed in this study. This data can be found at: National Intellectual Property Administration (CNIPA), CSMAR database and the China Research Data Service Platform (CNRDS). Data processing and analysis are conducted using Stata 18.
